# An Overlapping Cell Image Synthesis Method for Imbalance Data

**DOI:** 10.1155/2018/7919503

**Published:** 2018-07-09

**Authors:** Yi Ning Xie, Lian Yu, Guo Hui Guan, Yong Jun He

**Affiliations:** ^1^Harbin University of Science and Technology, Harbin 150080, China; ^2^Harbin Dongan Mitsubishi Automotive Engine Manufacturing Company, Harbin 150060, China

## Abstract

DNA ploidy analysis of cells is an automation technique applied in pathological diagnosis. It is important for this technique to classify various nuclei images accurately. However, the lack of overlapping nuclei images in training data (imbalanced training data) results in low recognition rates of overlapping nuclei images. To solve this problem, a new method which synthesizes overlapping nuclei images with single-nuclei images is proposed. Firstly, sample selection is employed to make the synthesized samples representative. Secondly, random functions are used to control the rotation angles of the nucleus and the distance between the centroids of the nucleus, increasing the sample diversity. Then, the Lambert-Beer law is applied to reassign the pixels of overlapping parts, thus making the synthesized samples quite close to the real ones. Finally, all synthesized samples are added to the training sets for classifier training. The experimental results show that images synthesized by this method can solve the data set imbalance problem and improve the recognition rate of DNA ploidy analysis systems.

## 1. Introduction

In recent years, cervical cancer with its incidence rate rising year by year has become a social problem which threatens women's lives. According to a survey report released by the World Health Organization in 2012, cervical cancer is the second largest killer of women in less developed areas [1, 2]. Cervical cancers can be detected at an early stage, and early diagnosis and early treatment are effective ways to deal with this problem. Currently, cervical smear is the most popular method for the screening of cervical cancer. In this method, human cervical exfoliated cells were first collected from patients and DNA contained in cells was stained. Then the stained specimen was placed under a microscope and observed by experienced pathologists to make a diagnosis. However, with the outbreak of cancers, this technique cannot meet the demand of practical applications. On the one hand, it requires great amounts of manpower and material resources; on the other hand, it often causes errors because of subjectivity and visual fatigue of pathologists. Therefore, automatic screening techniques become more and more important.

As an automatic screening technique, DNA ploidy analysis developed rapidly in recent years [3]. In this technique, cell specimens were first collected from patients and the DNA contained in cells was stained. Next, the specimens were placed under a microscope and images of the nucleus were taken using a high-resolution camera. Then, the nuclear images were classified and recognized by machine learning methods. Finally, the relative content of DNA in cells was measured and the abnormal cells were found to provide information for diagnosis. It is important for DNA ploidy analysis to analyze overlapping nuclei, where cancer cells are often found. However, it is difficult to collect enough overlapping nuclei images to learn a good classifier, because the samples of overlapping nuclei are few. Therefore, the number of overlapping nuclei images are far less than those of other categories, resulting in imbalance training data set problems [4, 5].

Most classifiers learned with imbalance data show poor performance when classifying samples from the classes with few training data. The samples from the minority classes are overwhelmed by those from the majority classes. Many methods have been proposed to solve this problem, and these methods can be mainly divided into two categories. The first category works at the data level, including resampling [6, 7] and feature selection approach [8]; the second one works at the algorithm level including the cost-sensitive [9] and single-class learning [10]. Resampling includes undersampling (removing samples from the majority class) and upsampling (creating new samples for the minority class). The most well-known method is the synthetic minority oversampling technique (SMOTE) [11] which interpolates among existing minority class examples and generates new minority class samples. But traditional SMOTE, which involves blindness, cannot solve the imbalance problems. Many improvements have been made by follow-up researchers, and some examples are SDSMOTE [12], GASMOTE [13], ECO-Ensemble [14], and WK-SMOTE [15]. At the algorithm level, the cost-sensitive learning-based method considers the costs associated with misclassifying samples, such as the cost-sensitive adaboost algorithm [16] and AdaCost [17]. Ensemble learning-based methods combine strength from individual learners and handle the class imbalance problem at the individual and ensemble levels, and some examples are the boosting algorithm [18, 19] and bagging algorithm [20, 21]. In addition, researchers also combine the resampling method with the learning algorithm method to deal with the class imbalance data sets, such as the PcBoost [22], CSFSG algorithm [23], HSDD method [24], and GADBSM method [25]. There are methods which use active learning to solve the class imbalance problem, such as the Bayesian active learning method [26] and the KA-SVM [27] method.

However, these methods can only learn from the existing samples, but cannot obtain class information beyond what is contained in the existing samples. For the imbalance data in cell classification, on the one hand, overlapping cells are formed by single cells; on the other hand, we can collect a large number of single-cell images easily. If we can simulate the process of generating overlapping images in the image data domain, we can generate enough overlapping cell images close to real images for feature extraction and model training. Therefore, we present a new method to synthesize overlapping nucleus images by using single-cell images.

In this paper, we present a new method to synthesize overlapping nucleus images by making use of the prior knowledge of forming overlapping nucleus images. This method first selects two-cell images and then synthesizes new overlapping nucleus images after rotation and segmentation. In order to make the synthesized cells as close as possible to the real ones, we consider three aspects in the proposed method. To ensure that synthesized cells are representative, we select typical single-cell images as source images. In order to avoid the excessive accumulation of the synthesized data, we introduce randomness for the rotation angle and the overlapping length for cells. To make the overlapping parts close to real ones, we reconstruct the pixels of the overlapping parts according to the Beer-Lambert Law [28, 29]. Experimental results show that after adding synthesized overlapping cell images to minority categories, the accuracy is improved on the three classifiers, including the multilayer perceptron (MLP, also called artificial neural network) [30], support vector machine (SVM) [31], and Gaussian mixture model (GMM) [32]. The proposed method also outperforms four typical methods (undersampling [33], upsampling [11], adaboost [34], and randomForest [35]) which are popular in solving the imbalance problem.

## 2. The Methods

As large amounts of single-cell images are available, we can synthesize two-cell images with two single-cell images; namely, three-cell images can be synthesized with a two-cell image and a single-cell image. Similarly, we can always synthesize a (*i* + *j*)-cell image with an *i*-cell image and a *j*-cell image.

**Figure 1:**
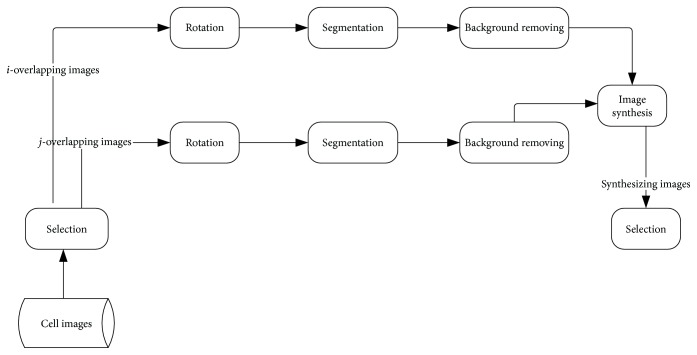
Synthesis scheme.

The procedure of image synthesis is shown in Figure 1. In the selection module, representative samples are chosen to avoid redundancy. Then the two selected images are rotated in a random angle, respectively. Next, two-cell images are segmented and the cell background is removed. Finally, the two segmented parts are overlapped to form an overlapping image, with the pixels of the overlapped part reconstructed according to the Beer-Lambert Law.

**Figure 2:**
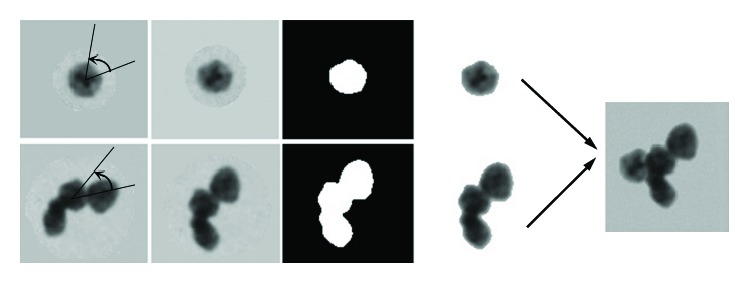
The synthesizing procedure.

The synthesizing procedure is shown in Figure 2. In order to obtain a 4-cell image, a single-cell image and a 3-cell image are chosen. After rotation, segmentation, and contour extraction, two-cell parts are overlapped to yield a new overlapping cell image.

### 2.1. Randomness Introduction

Randomness is employed to ensure the diversity of the generated overlapping cells. Firstly, rotation angles are randomly generated. Then, the overlapping length is random produced in an expected range. A uniform random number is generated by a linear random congruence method [36]. The basic recursive formula is presented as (1):

(1) $$\begin{array}{c} x_{n}=\alpha x_{n-1}+c\left(\mathrm{mod}M\right), \end{array}$$

*λ*_*n*_ = *x*_*n*_/*M*, *n* = 1, 2,…, where *x*_0_ is the initial value, *α* is the multiplier, *c* is the increment, and *M* is the modulus. They are all nonnegative integers.

### 2.2. Image Selection

Image selection [37] is aimed at ensuring that the selected images are representative. One feasible method is to prevent similar images from being used more than once. When selecting cell images to generate new overlapping images, representative samples which accurately reflect the larger entity should be chosen. In order to make the synthesized samples more representative, sample selection of cell images is necessary.  Algorithm 1 is used for image selection.where *n* is the feature dimension of the cell image. *P* is the initial sample set, while *Q* is the sample set after selection. *T* is the threshold value which is the mean distance of two samples in *P*, and *d*_*i*_ is the Euclidean distance of all the samples. *feature*(*α*) means the feature vector of the sample *α*.

### 2.3. Image Rotation and Segmentation

Image rotation refers to rotating an image with the centroid as center point. Given two-cell images, different overlapping cell images are generated when different rotation angles are used. The synthesized overlapping cell images can cover more conditions to ensure the diversity.

The original images for synthesis contain a background, which should be removed before synthesizing. In this paper, the threshold segmentation method is used to locate the cell area. In this method, pixels whose gray value is less than a threshold belong to the nucleus region; otherwise, the pixels belong to the background region. The segmentation formula is presented as (2):

(2) $$\begin{array}{c} F\left(x,y\right)=\left\{\begin{array}{c} 1,\;f\left(x,y\right)\geq T\\ 0,\;f\left(x,y\right)<T \end{array}\right., \end{array}$$

where *T* is the segmentation threshold, *f*(*x*, *y*) is a gray value in an image, and *F*(*x*, *y*) is the corresponding gray value after segmentation. The valley point of the histogram is set as the initial threshold.

**Figure 3:**
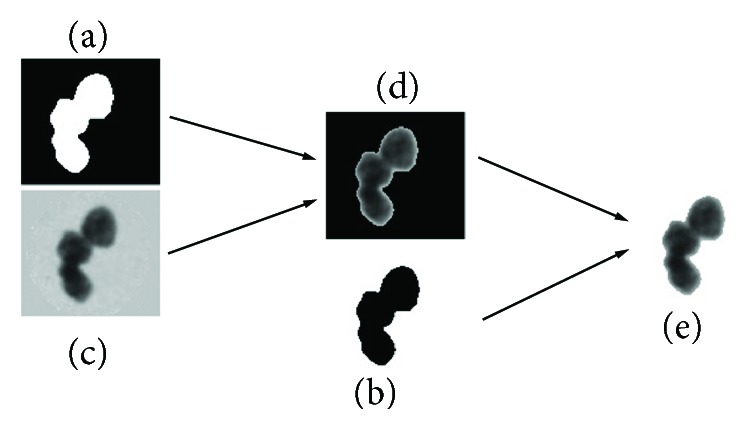
The process of removing background.

After image segmentation, nucleus contours are obtained. The cell region is extracted by removing the background of the cell image. This process is shown in Figure 3.

### 2.4. The Random Overlapping Length

**Figure 4:**
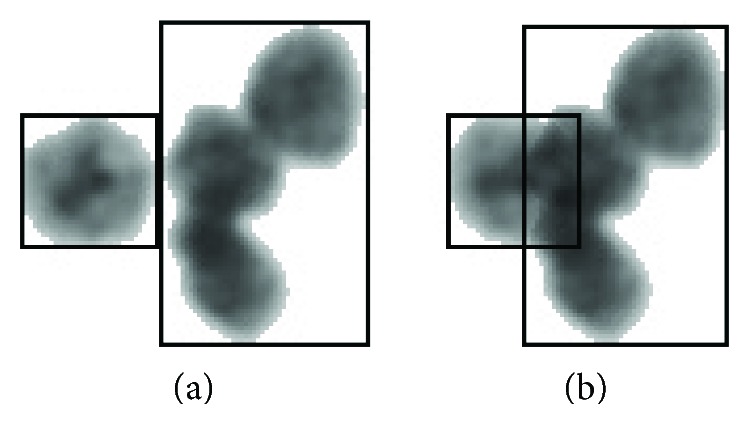
The random overlapping results.

The overlapping cells have a common area. We use overlapping length to describe the degree of overlapping. When one nuclear region is tangent to the second nuclear region (as shown in Figure 4(a)), the value of *d* is zero. Here, the overlapping length is subject to 0 ≤ *d* ≤ 1/2*R*_min_ (*R*_min_ refers to the minimum width value of two nuclear regions). The overlapping length of two black rectangles (as shown in Figure 4(b)) is a random value generated by (1).

### 2.5. Pixel Reconstruction of Overlapping Regions

**Figure 5:**
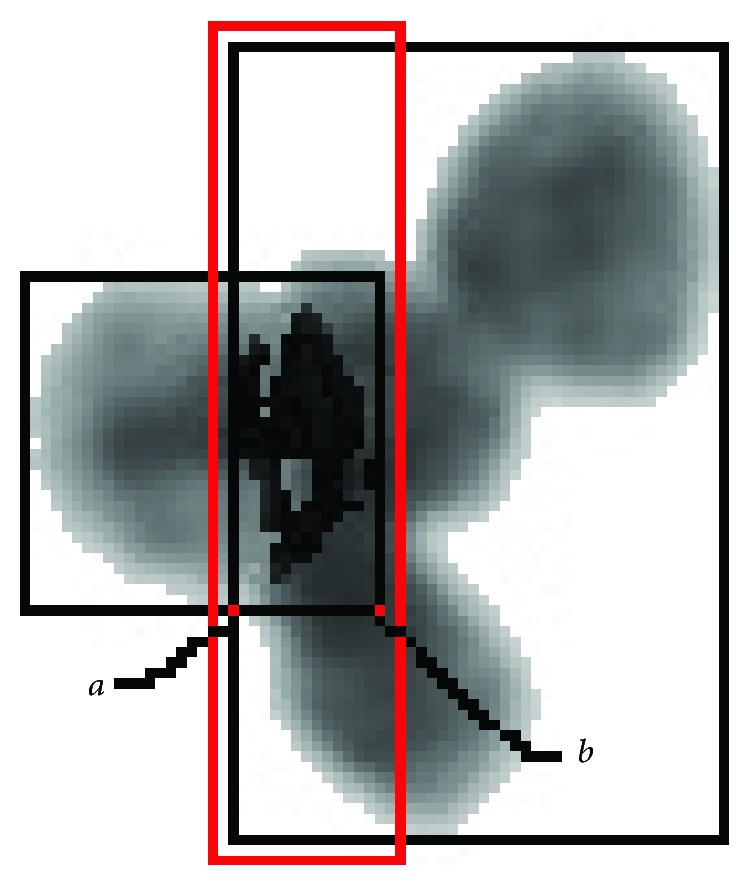
Nuclei overlapping region.

The nonoverlapping regions of cells remain unchanged after the overlapping operation. However, the overlapping region is too dark, which is not in accordance with the real cell images. Therefore, it is necessary to reconstruct the gray value of the overlapping region. First of all, we need to locate overlapping areas. The specific steps are as follows:

1. Finding the smallest cross *x* and vertical coordinates *y*, the maximum horizontal *X* and vertical coordinates *Y*, according to the coordinates of all points in the cell areas, and point (*x*, *y*) and (*X*, *Y*) respectively, are coordinates of the upper left and lower right corner of the minimum bounding rectangle in the nuclear region. In the same way, the minimum bounding rectangle is obtained (such as the two black rectangles in Figure 5), and two rectangles are intersecting at the point of *a* and *b* (as points *a* and *b* in Figure 5).
2. The length of *ab* with the added 2 points is the width, and the rectangle's height is the new height; with these, a new searching area is constructed (such as the red rectangle in Figure 5).
3. Every pixel in the searching area of the nonwhite part is traversed. If this point is within the first contour and at the same within second one, this point is determined as the one that need be reconstructed.
4. All pixels that need to be reconstructed are searched, and all the points form a reconstruction pixel set.
5. The pixel in the reconstruction pixel set is given a new value via (7).

**Figure 6:**
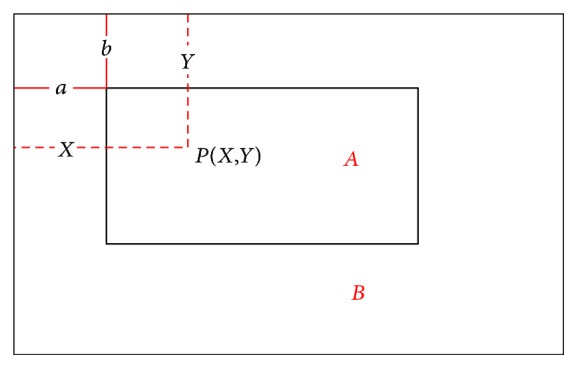
Coordinate conversion.

Since the two pictures are operated in one background image, the positions in the background and in the source image need a coordinate transformation. As is shown in Figure 6, assuming that the background is rectangle *B*, the source image is rectangle *A*. (*X*, *Y*) is the position of point *P* in *B*, (*a*, *b*) is the position of point *P* in *B*, and (*x*, *y*) is the position of point *P* in *A*. The formula used for position transformation is presented as (3):

(3) $$\begin{array}{c} \begin{array}{c} x=X-a,\\ y=Y-b. \end{array} \end{array}$$

With (3), the point coordinates are obtained in the overlapping region of the source image, then the corresponding pixel values can be obtained.

According to the Beer-Lambert Law [24, 25], we can infer the pixel gray value in the overlapping areas. Firstly, a gray value of the point is converted to the value of optical density, and then optical density is accumulated. Finally, the value of optical density is converted to grayscale values. The gray value cannot be added directly in overlapping cell images. Since the absorbance represents the amount of cellular materials, the absorbance of the overlapping part can be superimposed. Therefore, the gray value of the overlapping region needs the conversion process. For the two overlapping cells, the relationship between gray values and the optical density can be modeled as follows:

(4) $$\begin{array}{c} A_{1}=\mathrm{lg}\left(\frac{I_{0}}{I_{1}}\right), \end{array}$$

(5) $$\begin{array}{c} A_{2}=\mathrm{lg}\left(\frac{I_{0}}{I_{2}}\right), \end{array}$$

where *I*_0_ is the average gray value of the background (*I*_0_ is the threshold), *I*_1_ denotes a gray value in the first cell, and *I*_2_ are the gray values in the second cell. *A*_1_ and *A*_2_ are the corresponding optical density values.

When the two points in the two cells are overlapping, the optical density satisfies the following additive relation:

(6) $$\begin{array}{c} A=A_{1}+A_{2}=\mathrm{lg}\left(\frac{I_{0}}{I_{1}}\right)+\mathrm{lg}\left(\frac{I_{0}}{I_{2}}\right)=\mathrm{lg}\left(\frac{I_{0}I_{0}}{\left(I_{1}I_{2}\right)}\right)=\mathrm{lg}\left(\frac{I_{0}}{\left(I_{1}I_{2}/I_{0}\right)}\right)=\mathrm{lg}\left(\frac{I_{0}}{I_{s}}\right), \end{array}$$

where *A* is the new optical density of the corresponding position at an overlapping point, and *I*_*s*_ is the new gray value at the overlapping point. According to (6), the new gray value can be computed with

(7) $$\begin{array}{c} I_{s}=\frac{I_{1}I_{2}}{I_{0}}. \end{array}$$

**Figure 7:**
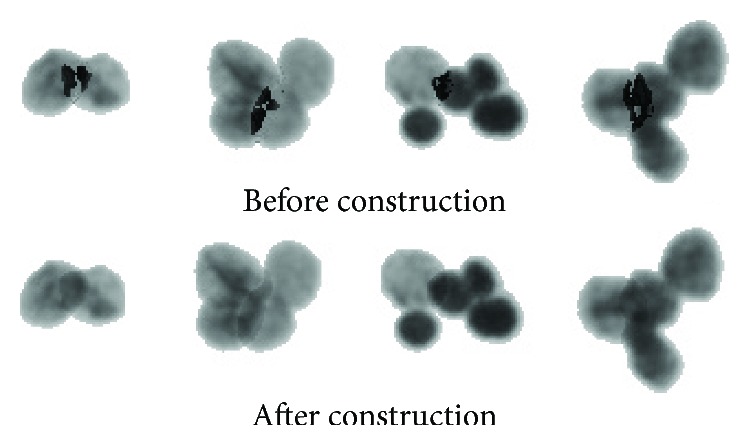
Comparison of constructing overlapping parts.

As shown in Figure 7, it can be seen that the synthesized overlapping region is darker than the real overlapping region. After reconstruction, the overlapping region looks more natural.

## 3. The Results

### 3.1. Experiments

A DNA ploidy analysis system is mainly used for the identification and analysis of diseased cells and cancer cells. In order to obtain real data, the samples are collected by the staffs of the Heilongjiang Maria Maternity Hospital. The cell samples were collected from 300 patients. The cells of each patient were smeared on a slide and then Feulgen stained. After that, the slide was placed under a microscope and the microscope automatically took cell images. Then, the DNA ploidy analysis system segmented cell images into single-cell images or overlapping cell images. Finally, cell pathology doctors classified each cell image manually into one of 8 categories, namely, single typical epithelial cell, single atypical epithelial cell, two epithelial cells, three epithelial cells, four or more epithelial cells, single lymphocyte, single centriole, and two or more centrioles. These cell images of each class are examples of a typical imbalance. The amount of single-cell images in classes 1, 2, 3, 4, and 6 are very large, while those of the other classes are very small. Our task is to synthesize overlapping samples for classes 4, 5, 7, and 8 with single-cell images. First of all, we need to select representative samples from classes 1, 2, 3, and 6. The cells in classes 1, 2, 3, 4, and 6 are used to synthesize new overlapping images, and the images of these classes need sample selection. The original data in the experiment are extremely unbalanced. In order to show the influence of the imbalance data on the accuracy rate, the number of testing samples is 2000 in each class. There are 8 types of cell images in total, and classes 3, 4, 5, and 8 have small number of training samples. Experiments are performed by adding synthesized cell data to these classes (i.e., classes 3, 4, 5, and 8) to make the data more balanced. In the experiments, the synthesized data are added into the training set gradually to make it more and more balanced.

Three popular classifiers, that is, the multilayer perceptron (MLP), support vector machine (SVM) and mixed Gaussian model (GMM), are chosen to evaluate the proposed method. The classifiers are trained with the new train sets of different amounts and their performance is compared. In the neural network training, the hidden node is 100, and the number of iterations is 200. The minimum error in training is set as 0.1. The number of transformation characteristics is 5. The random seed value is 20 to initialize the multilayer perceptron. In the SVM classifier, the number of transformation parameters is 80. Kernel type is rbf, and the mode of the classifier is one-versus-one. In the Gaussian model classifier, the pretreatment type is a normalization and pretreatment parameter (the number of transformation characteristics) with a value of 100, which is used for its transforming characteristics. The seed value generated by the randomizer is 42.

### 3.2. Feature Extraction

Based on the features of cell images, 45 dimensional features are first extracted and then 28 dimensional features are selected for classification. The selected features include 20 morphologic features [38] and 8 texture features [39]. They are essential for distinguishing 8 types of cell images in classification. The 20 morphologic features are used to describe the shape and size of cells, including area, circularity, distance, sigma, sides, roundness, convexity, *I_a_* (centroid coordinates of *x* axis), *I_b_* (centroid coordinates of *y* axis), *M*_11_, *M*_02_, *M*_20_, compactness, ContLength, diameter, radius, rectangularity, anisotropy, bulkiness, and StructureFactor [38]. The 8 text features consist of contrast, energy, homogeneity, correlation, entropy, anisotropy, mean, and deviation [39]. Some typical morphologic features can be defined by (8), (9), (10), (11), (12), (13), (14), and (15), and two typical texture features, that is, the mean and deviation can be expressed by (14) and (15).

(8) $$\begin{array}{c} Sigma=\sqrt{\underset{x,y}{\sum }{\left(\left\|g_{0}-g_{\left(x,y\right)}\right\|-distance\right)}^{2}/area,} \end{array}$$

(9) $$\begin{array}{c} Distance=\underset{x,y}{\sum }\frac{\left\|g_{0}-g_{\left(x,y\right)}\right\|}{area}, \end{array}$$

(10) $$\begin{array}{c} Roundness=\frac{1-sigma}{distance}, \end{array}$$

where *g*_0_ represents the mean values of pixels of the cell area, and *g*_(*x*, *y*)_ is the pixel value of dot (*x*, *y*) in the area of the cell.

(11) $$\begin{array}{c} h=\frac{M_{20}+M_{02}}{2}, \end{array}$$

(12) $$\begin{array}{c} I_{a}=h+\sqrt{h^{2}-M_{20}*M_{02}+M_{11}^{2}}, \end{array}$$

(13) $$\begin{array}{c} I_{a}=h-\sqrt{h^{2}-M_{20}*M_{02}+M_{11}^{2}}, \end{array}$$

where *M*_20_ and *M*_02_ mean the sum of the pixel values of the *x* and *y* axes of the nucleus separately. *M*_11_ is the mean value of every pixel in the nucleus.

(14) $$\begin{array}{c} Mean=\frac{\sum_{x,y}g\left(x,y\right)}{Num}, \end{array}$$

(15) $$\begin{array}{c} Deviation=\sqrt{\sum_{x,y}{\left(g\left(x,y\right)-mean\right)}^{2}/Num}, \end{array}$$

where *g*(*x*, *y*) is the gray values of the pixel(*x,y*) and Num is the number of pixels of images.

For each cell image, the 28 features extracted for classification are shown in Table 1.

### 3.3. Evaluation Criteria

For multiple class problems, we suppose that the classes have been labelled *C*_0_, *C*_1_, *C*_2_,…, *C*_k_ (*k* > 2) with the order of the labels which do not reflect any intrinsic order to the classes. The results of classifications are accessed according to the confusion matrix shown in Table 2. Their total accuracy is computed via (16). The recall rate of each class is computed in (17), and the G-mean can be computed via (18).

(16) $$\begin{array}{c} Accuracy=\frac{n_{11}+n_{22}+\cdots +n_{kk}}{\sum_{i=1}^{k}n_{i1}+n_{i2}+\cdots +n_{ik}}, \end{array}$$

(17) $$\begin{array}{c} {Recall}_{i}=\frac{n_{ii}}{\sum_{j=1}^{k}n_{ij}}, \end{array}$$

(18) $$\begin{array}{c} G‐mean={\left(\overset{k}{\underset{i=1}{\prod }}{recall}_{i}\right)}^{1/k}, \end{array}$$

where *C*_*i*_ represents the label of the class *i*, and *n*_*ii*_ means that the sample number from class *i* is predicted to be class *i* in (18). *k* is more than 2 in these equations.

### 3.4. Results

The image number for training in each class is shown in Table 3. The synthesized cell images are added to the training data to make them more balance. The accuracy of the three classifiers is compared in Table 4, where the conditions, imbalance ratio (the ratio of the number of samples of the largest class to the smallest class), the accuracy rate, and G-mean are shown and the experimental results of the inadequate training and full training with synthesized cells are compared. The entries in the table are sorted by descending order on an unbalanced ratio, namely the training data become balanced gradually.

**Figure 8:**
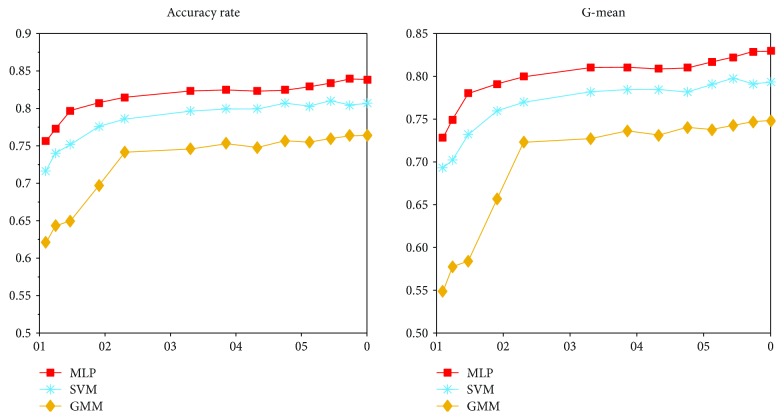
The results in the range of distribution ratio of a small class.

As shown in Table 4, when the imbalance ratio is 100, the original data without adding synthesized samples are used for training. The accuracy rates obtained by these three classifiers are the lowest. With the imbalance ratio decreasing, the accuracy increases. When the unbalance ratio is 1, that is, the sample numbers of all classes become the same, the three classifiers achieve their best performance compared with 1, and the accuracy rates are increased by 8.29%, 8.97%, and 14.34%, respectively; at the same time the G-mean reaches 0.8292, 0.7931, and 0.7484, respectively. The accuracy rate and G-mean changes, respectively, in the range of the distribution ratio of a small class in Figure 8.

### 3.5. Comparison with Other Methods

Four methods, namely, the proposed method, upsampling [11], undersampling [33], and the adaboost method [34] are compared. The proposed method can be treated as an upsampling method which simulates the process of generating overlapping images in the image data domain. In the upsampling method, new features in feature space-based SMOTE [11] are generated. In the undersampling method, the training data are divided into clusters. Then, in view of the ratio of majority class samples to minority class samples, the representative data for majority class samples from each cluster are selected. Adaboost is an iterative algorithm, which places different weights on the training distributions in each iteration. After each iteration, the classifier increases the weights associated with the incorrectly classified examples and decreases the weights associated with the correctly classified examples separately. This forces the learner to focus more on the incorrectly classified examples in the next iteration.

**Figure 9:**
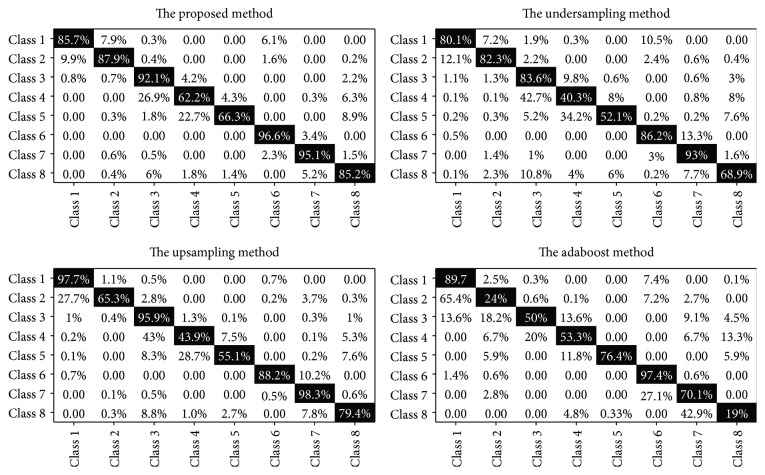
The comparison of the confusion matrix by 4 single methods.

The proposed method, the undersampling method, and the upsampling method use the MLP classifier, while the adaboost method uses the adaboost algorithm. In the adaboost classifier, the number of iterations is 50 and the learning rate is 1.0. The confusion matrix can show the relationship between the predicted results and the original cell classes. The assessment results on the precision of the classification using the confusion matrix comparing with 4 methods are shown in Figure 9.

As can be seen from Figure 9, in the proposed method, three epithelial cells (class 4) and four or more epithelial cells (class 5) have a lower accuracy rate of 62.2% and 66.3%, respectively. In comparison, the accuracy rate of class 4 and class 5 is only 40.3% and 52.1% in the undersampling method, 43.9% and 55.1% in the upsampling method, and 53.3% and 76.4% in the adaboost method. The images of class 4 and class 5 are difficult to classify because of diverse overlapping situations and overlapping cell numbers. In conclusion, the proposed method achieves the best performance, while the adaboost method gets the worst performance.

According to literature, when the resampling method combines with the learning algorithm, good performance can be obtained. Therefore, we choose the randomForest algorithm [35] to train models. The randomForest belongs to an ensemble learning method, which fits a number of decision tree classifiers on various subsamples of the data sets. It also uses an averaging value to improve the predictive accuracy and control overfitting. We combine the upsampling method with the randomForest method, the proposed method with the adaboost method, and the proposed method with the randomForest method. In the randomForest classifier, the number of iterations is 60, the maximum depth of each tree is 3, the minimum number of sample leaves is 20, and the maximum features is “sqrt.”

**Figure 10:**
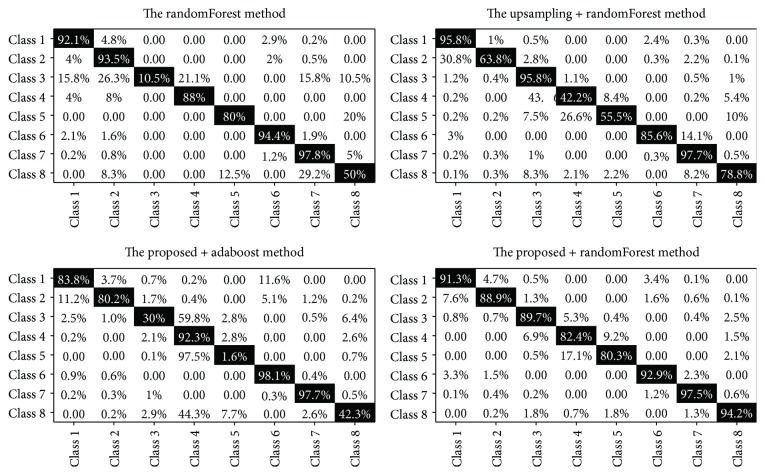
The comparison of the confusion matrix by 4 hybrid methods.

As can be seen from Figure 10, the combinations of the two methods have a higher accuracy than that of a single method. The accuracy rate of class 3 is only 10.5%, which is extremely abnormal in the randomForest method, and the accuracy rate of class 8 just reaches 50%, relatively low compared to the other 6 classes except class 3. However, in the upsampling + randomForest method, the accuracy rate of class 3 obtains an accuracy of 95.8% and the accuracy rate of class 8 is 78.8%. According to the confusion matrix of the proposed + adaboost method, this method is not suited to deal with the balanced data generated by the proposed method. Finally, in the proposed + randomForest method, the accuracy rate of each class is good, and the lowest accuracy among 8 classes is 80.3%. Therefore, the proposed + randomForest method achieves the best performance among the 4 hybrid methods.

**Figure 11:**
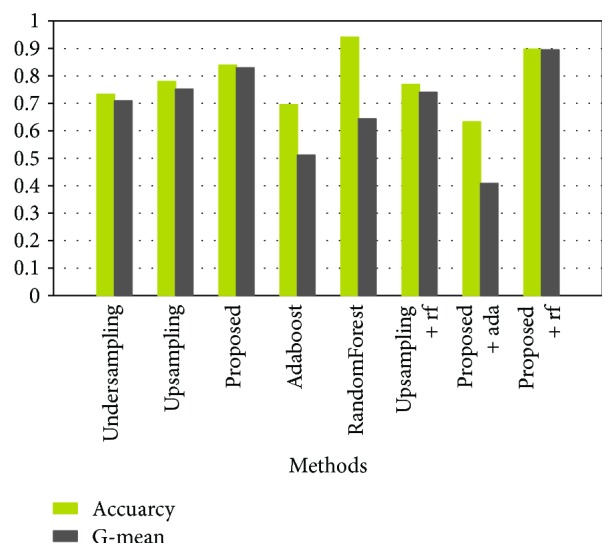
The comparison of 8 methods (ada means the adaboost method, rf refers to the randomForest method).

Even though the confusion matrix can indicate the accuracy rate of each type of cells in detail, it cannot directly show the overall correctness, G-mean, and so on.  Figure 11 shows the results of all 8 methods.

As can be seen from Figure 11, the accuracy of the randomForest method is the highest, but the G-mean is far from the value of accuracy. It is obvious that the randomForest method is not suitable for dealing with imbalance data and it pays more attention to the samples of the majority class and ignores the samples of the minority class. Therefore, the method proposed effectively solves the imbalance problem. As for the proposed + randomForest method, the accuracy is close to that of G-mean, while they are higher than those of the other methods except for the randomForest method. The accuracy and G-mean of the proposed method are less than that of the proposed + randomForest method. The accuracy is high but the G-mean is relatively low in the proposed + adaboost method, so it also performs worse on the imbalance data. In summary, judging by all the evaluation criteria, the proposed + randomForest method has achieved the best performance.

In fact, the classifier can perform better by adjusting the parameters of the learning algorithm.  Table 5 shows the range of results when parameters are varied. The data used in the adaboost and randomForest are synthesized by the proposed method.

As can be seen from Table 5, in the adaboost algorithm, the accuracy and G-mean decrease in a trend when the iteration number increases. When the iteration number is 0.4, the accuracy is the highest. However, the algorithm can cause more errors when the learning rate is low. When the iteration number is 80 and the learning rate is 0.8, the classifier performs its best. The accuracy and G-mean of the randomForest method show an upward tendency with the iteration number increasing from 10 to 60. The accuracy decreases when the iteration number increases from 60 to 80. When the iteration number is 100, the randomForest algorithm performs its best, because the values of the accuracy and G-mean are both relatively high.

## 4. Conclusion

In conclusion, we proposed a new method to synthesize overlapping cell images to solve the imbalance data problem. This method simulates the generation of overlapping cells by making use of prior knowledge. In this method, representative images are first chosen, and then the images are rotated randomly. After that, two segmented cell parts are overlapped, and finally the overlapping parts are reconstructed. Sample selection and randomness are introduced to make the synthesized images more representative. The new images are added to the training samples for model training. Experiments show that the proposed method greatly improves the accuracy of cell classification. The accuracy is improved from 75.58% to 83.93% and G-mean is improved from 0.7280 to 0.8292. When we combine the synthesized method with the randomForest algorithm, the accuracy reaches around 89.7% and the G-mean gets about 0.895. With the proposed method, a large amount of images can be generated. It is an interesting topic to select synthesized samples according to the performance of classification. In the future, we will focus on the task to select representative synthesized samples with the active learning method.

## Figures and Tables

**Algorithm 1:**
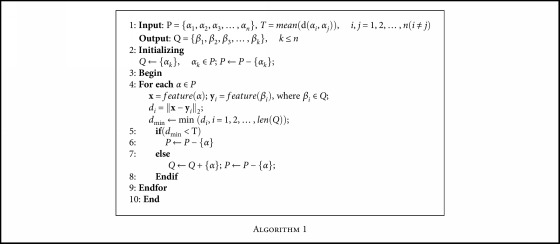


**Table 1:** Features of each class of cell images.

Class	1	2	3	4	5	6	7	8
Cell images	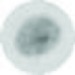	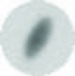	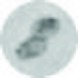	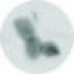	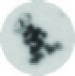	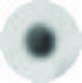	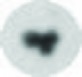	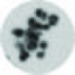
IOD	89.610977	71.606436	180.15888	272.85886	470.77552	76.777616	86.473958	940.99801
Area	837	447	1341	1705	1805	289	299	2910
Circularity	0.7921	0.480011	0.461493	0.382338	0.479377	0.920065	0.654806	0.510143
Roundness	0.903653	0.727129	0.68578	0.640621	0.732710	0.944292	0.812886	0.722639
Radius	18.718845	17.566048	30.722118	37.70215	33.625638	10.377715	12.087826	41.920397
Deviation	0.056678	0.096081	0.080454	0.090358	0.173582	0.159820	0.180099	0.18441
Mean	0.642317	0.57354	0.6061	0.573193	0.482879	0.470737	0.457682	0.430258
Sigma	1.475920	3.135578	6.373434	8.491296	6.312004	0.474985	1.634735	8.020041
Contrast	1.593787	6.369128	2.674124	2.777126	9.373961	16.356402	15.341137	12.716838
Convexity	0.974389	0.959227	0.90303	0.830088	0.827602	0.969799	0.934375	0.812395
Bulkiness	1.002132	1.001983	1.051246	1.223468	1.161857	1.000329	1.045995	1.132826
StructureFactor	0.283446	1.125497	1.343892	1.689330	0.973999	0.049896	0.463667	0.815918
*I*_*a*_	71551.46	33796.029	335417.10	622132.67	511789.63	6978.0203	10412.977	1223692.3
*I*_*b*_	43622.82	7510.4405	67471.275	128759.63	177297.55	6334.6855	5318.0129	476218.75
*M*_11_	−0.018070	−0.054505	−0.073719	0.084856	0.049427	0.001029	0.007442	−0.039142
*M*_20_	0.073786	0.140186	0.122783	0.129873	0.119612	0.083408	0.060474	0.120763
*M*_02_	0.073786	0.066543	0.101258	0.128430	0.091892	0.075985	0.115486	0.079980
Energy	0.023546	0.008648	0.011273	0.011569	0.003225	0.004424	0.005000	0.002741
Correlation	0.941807	0.920241	0.950388	0.959477	0.962070	0.925055	0.943140	0.954548
Homogeneity	0.601926	0.313502	0.511144	0.515849	0.315213	0.239775	0.322485	0.306934
Entropy	5.706993	6.248901	6.224919	6.415742	7.111077	6.711657	6.795570	7.259227
Anisotropy	−0.525847	−0.510334	−0.537583	−0.514221	−0.514942	−0.490626	−0.499718	−0.499788
Compactness	1.108596	1.311802	1.633726	1.912168	1.913056	1.028384	1.198749	2.272140
ContLength	107.9827	85.840620	165.92388	202.40916	208.30865	61.112698	67.112698	288.24978
Diameter	36.359318	34.132096	60.440053	74.404301	64.412732	19.646883	23.021729	82.800966
Rectangularity	0.801250	0.800937	0.793462	0.646409	0.707792	0.804348	0.771812	0.678742
Distance	15.318871	11.491077	20.283375	23.627692	23.614855	8.526281	8.736569	28.915494
Sides	4.261805	2.606224	2.438174	2.288309	2.631792	5.520584	3.114689	2.586205
NumRuns	32.000000	32.000000	52.000000	62.000000	67.000000	20.000000	18.000000	91.000000
MeanLength	26.156250	13.968750	25.788462	27.500000	26.940299	14.450000	16.611111	31.978022

**Table 2:** Confusion matrix for multiple class classification problems.

		Predicted classes			
		*C*_1_	*C*_2_	…	*C*_*k*_
Actual classes	*C*_1_	*n*_11_	*n*_12_	…	*n*_1*k*_
	*C*_2_	*n*_21_	*n*_22_	…	*n*_2*k*_
	…	…	…	…	…
	*C*_*k*_	*n*_*k*1_	*n*_*k*2_	…	*n*_*kk*_

**Table 3:** The number of cell images with different conditions.

Classes								Imbalance ratio	Conditions
1	2	3	4	5	6	7	8		
20,000	20,000	200	200	200	20,000	20,000	200	100.0	1
20,000	20,000	500	500	500	20,000	20,000	500	40.0	2
20,000	20,000	1000	1000	1000	20,000	20,000	1000	20.0	3
20,000	20,000	2000	2000	2000	20,000	20,000	2000	10.0	4
20,000	20,000	4000	4000	4000	20,000	20,000	4000	5.0	5
20,000	20,000	6000	6000	6000	20,000	20,000	6000	3.3	6
20,000	20,000	8000	8000	8000	20,000	20,000	8000	2.5	7
20,000	20,000	10,000	10,000	10,000	20,000	20,000	10,000	2.0	8
20,000	20,000	12,000	12,000	12,000	20,000	20,000	12,000	1.7	9
20,000	20,000	14,000	14,000	14,000	20,000	20,000	14,000	1.4	10
20,000	20,000	16,000	16,000	16,000	20,000	20,000	16,000	1.3	11
20,000	20,000	18,000	18,000	18,000	20,000	20,000	18,000	1.1	12
20,000	20,000	20,000	20,000	20,000	20,000	20,000	20,000	1.0	13

**Table 4:** The results with the range of conditions.

Conditions	Imbalance ratio	Accuracy (%)			G-mean		
		MLP	SVM	GMM	MLP	SVM	GMM
1	100.0	75.58	71.68	62.05	0.7280	0.6932	0.5486
2	40.0	77.24	74.02	64.29	0.7496	0.7021	0.5776
3	20.0	79.73	75.18	65.01	0.7799	0.7318	0.5841
4	10.0	80.77	77.61	69.73	0.7912	0.7598	0.6570
5	5.0	81.49	78.57	74.15	0.7994	0.7696	0.7229
6	3.3	82.33	79.64	74.58	0.8102	0.7820	0.7272
7	2.5	82.47	79.92	75.28	0.8106	0.7845	0.7363
8	2.0	82.30	79.93	74.79	0.8086	0.7846	0.7314
9	1.7	82.43	80.70	75.69	0.8097	0.7816	0.7404
10	1.4	82.88	80.31	75.49	0.8167	0.7903	0.7379
11	1.3	83.38	81.03	75.98	0.8225	0.7979	0.7427
12	1.1	83.93	80.47	76.33	0.8290	0.7913	0.7470
13	1.0	83.87	80.65	76.39	0.8292	0.7931	0.7484

**Table 5:** The results with the range of parameters in a classifier.

Adaboost		Accuracy	G-mean	RandomForest	Accuracy	G-mean
Iteration number	Learning rate			Iteration number		
50	1	0.6327	0.4080	10	0.8016	0.7885
80	1	0.6485	0.5595	30	0.8142	0.8022
100	1	0.6354	0.5609	40	0.8977	0.8951 0.8935
120	1	0.5902	0.5255	50	0.8935	0.8914
150	1	0.5849	0.4602	60	0.8973	0.8948
80	0.8	0.7045	0.6241	80	0.8928	0.8894
80	0.7	0.7406	0.6214	100	0.8940	0.8916
80	0.6	0.7232	0.5777	120	0.8249	0.8157
80	0.5	0.7682	0.6260	150	0.8949	0.8923
80	0.4	0.7767	0.6601	200	0.8937	0.8910

## Data Availability

The data used to support the findings of this study are available from the corresponding author upon request.
